# A Clinical Audit of the Use of CT Head Scan Post-Inpatient Falls in Hospitalised Older Adults Utilising a Post-Fall Clinical Pathway

**DOI:** 10.3390/ijerph22071098

**Published:** 2025-07-11

**Authors:** Isabel Watt, Ruth Devin, Joyita Bhattacharya, Frances Waldie, Emma Holden, Chiung-Jung (Jo) Wu

**Affiliations:** 1 Northern Adelaide Local Health Network, Adelaide, SA 5000, Australia; isabel.watt@uqconnect.edu.au; 2 Sunshine Coast Hospital and Health Service, Birtinya, QLD 4575, Australia; ruth.devin@health.qld.gov.au (R.D.); joyita.bhattacharya@health.qld.gov.au (J.B.); frances.waldie@gmail.com (F.W.); 3 Metro North Hospital and Health Service, Brisbane, QLD 4029, Australia; emma.holden2@health.qld.gov.au; 4 School of Health, University of the Sunshine Coast, Petrie, QLD 4502, Australia

**Keywords:** inpatient falls, older adults, head injury, CT scan, clinical audit

## Abstract

**Background:** Older adults are at high risk of falls, and head injuries following these events can have devastating consequences. The In-Patient Post Fall Clinical Pathway is a tool utilised in many hospitals in Queensland, Australia, to guide the need for CT brain imaging post-inpatient fall. This audit aimed to assess the use of CT imaging following inpatient falls in older adults, evaluate adherence to the In-Patient Post Fall Clinical Pathway, and explore factors associated with serious head injury. **Methods:** A retrospective audit was conducted across two regional Queensland hospitals over 2.5 years. Falls involving patients aged over 65 years were included. Data were analysed using descriptive and bivariate statistical tests. **Results:** Among 874 eligible falls, the mean patient age was 80.4 years, and approximately two-thirds were male. While 90.6% of patients who had fallen met clinical pathway criteria for a CT head scan, only 50.1% of them received a scan. Serious head injuries were uncommon (2.25% of total falls), with subdural haematoma being most frequent. Only one patient underwent neurosurgical intervention. No missed serious injuries were identified. No individual characteristic was significantly associated with serious head injury, although trends were observed for unwitnessed falls, falls from bed, falls with a head strike, new symptoms four hours post-fall, and anticoagulant use. **Conclusions:** There is a gap between clinical pathway recommendations and imaging practices, with clinicians often relying on judgement over strict adherence to guidelines. Further research is needed to inform evidence-based and practical decision-making to balance imaging use with clinical risk.

## 1. Introduction

The World Health Organization (WHO) defines falling as the act of inadvertently coming to rest on the ground or other lower level [1]. Falls are associated with significant morbidity and mortality and are costly, both on an individual and healthcare system level [1,2,3]. In Australia, falls are the leading cause of unintentional injury death [2]. Although there is substantial literature on community falls, there is a relative paucity on those that occur in the inpatient setting. Inpatient falls are common, particularly among the geriatric population [2]. Indeed, inpatient falls are the leading cause of preventable harm among hospitalised older adults [4]. Australian data from 2018 reported seven injurious falls per 1000 public-sector hospitalisations in adults aged 65 years and older, which increased to 17.6 injurious falls per 1000 hospitalisations among those aged over 85 years [2].

Falls can result in a variety of injuries, including bony and soft tissue injuries [2]. Arguably, the most important injuries are acute, serious head injuries, specifically traumatic intracranial haemorrhage and skull fracture [5]. These can be disabling or life-ending and at times require urgent neurosurgical intervention to prevent further harm [5]. Acute, serious head injuries can be detected on computerised tomography (CT) head scan [6]. However, the decision to proceed with a scan to look for such injuries needs to be balanced with the cost and risks of the scans themselves. This is particularly apparent if a patient requires sedation or must be transferred to another hospital to facilitate CT. In the climate of “Choosing Wisely” and “Evolve” [7,8], we aim to reduce low-value healthcare and make better use of resources. Clinicians want to ensure that the people who are receiving scans are the ones who are going to benefit from them. Is this faller at risk of an acute serious head injury? Is a scan going to change their management?

Many clinical tools exist to help guide the decision as to which patients should receive a CT head scan after a fall. These tools are well validated for the emergency and outpatient setting and include examples such as the NICE guidelines [9], the Canadian CT Head Rules, and the New Orleans Criteria [6]. These guidelines identify characteristics associated with an increased risk of acute, serious head injury and recommend scanning those with high-risk features present. Examples of characteristics warranting imaging per these guidelines include the following: a dangerous mechanism of injury, a reduced Glasgow Coma Scale (GCS), signs of skull fracture, post-traumatic seizures, focal neurological signs, nausea and vomiting, retrograde amnesia for longer than 30 min, a history of bleeding or clotting disorders, or taking anticoagulation medication [6].

Conversely, there is a relative scarcity of evidence and guidelines for the inpatient setting, and most inpatient tools are based on the extrapolation of data from the emergency and outpatient spheres [6]. The In-Patient Post Fall Clinical Pathway is one such tool utilised in many hospitals in Queensland [10]. The In-Patient Post Fall Clinical Pathway was first developed in 2010, and it was most recently revised in 2023 [10]. It recommends a CT head scan post-inpatient fall if any of the following features are present: a fall from greater than one metre in height (i.e., high-risk mechanism of injury), any unwitnessed fall, a suspected head injury, a recent surgery or procedure, known coagulopathy, the current receipt of anticoagulant or antiplatelet therapy, neurological observations, or any concerning symptoms (e.g., behavioural change, headache, and vomiting) in the 24 h following the fall.

### Aims

This study aimed to assess the use of CT head scans following inpatient falls among older adults in two regional hospitals in Queensland, Australia. Specifically, we examined adherence to the In-Patient Post Fall Clinical Pathway, which was reported to be inconsistently applied in clinical practice. Additionally, we sought to determine the prevalence and characteristics of acute, serious head injuries sustained among hospitalised people who fell and to identify whether specific clinical features were associated with increased risk. The findings from this study may help clinicians more accurately identify patients most likely to benefit from CT imaging and inform potential revisions to the In-Patient Post Fall Clinical Pathway.

## 2. Materials and Methods

### 2.1. Study Design, Participants, and Setting

A retrospective review was conducted using data from medical records and hospital systems of two regional hospitals in Queensland, Australia (Sunshine Coast University Hospital and Nambour General Hospital), covering a 2.5-year period (November 2017 to July 2020).

The inclusion criteria were patients aged 65 years and older admitted to the study sites who experienced a fall during the study period. While the WHO considers 60 years as the beginning of older adulthood, a cutoff of 65 years was determined to align with local clinical practice and standard admission criteria for geriatric care in Australia. Exclusion criteria were episodes not meeting the WHO definition of a fall (i.e., “near misses”) [1]; episodes where patients were located in the emergency department, the short stay unit, the transit lounge, day units (chemotherapy, dialysis, and infusion clinics), the mental health unit, and the intensive care unit; and episodes where patients were admitted to the “Hospital in the Home” programme. These admission types were excluded, as they were felt to either not represent the desired inpatient cohort (i.e., emergency department, short stay unit, transit lounge, day unit, and “Hospital in the Home” admissions) or because they use differing documentation processes (i.e., mental health and intensive care unit admissions).

### 2.2. Data Collection

Eligible fall events were identified using hospital incident management software (RiskMan and Prime), followed by a review of electronic medical records. The data collected included demographic information, comorbidities, length of stay, readmission rates, whether patients met the In-Patient Post Fall Clinical Pathway criteria to have a CT head scan performed, whether a CT head scan was actually administered, the injuries identified and their management, and mortality outcomes. Data on non-head injury fractures, which were explored in a separate publication [11], were also collected. Five trained data collectors used a medical audit tool to ensure consistency [12].

### 2.3. Statistical Analysis

All analyses were conducted using the IBM Statistical Package for the Social Science, version 27, with a pre-defined level of statistical significance of *p* < 0.05. Sample characteristics for categorical data were examined using frequencies (e.g., sex, recurrent fall event, witnessed/unwitnessed, fall height, head strike, etc.). The Mann–Whittney *U* test was used to examine the relationships between non-normally distributed continuous variables and categorical variables (e.g., age and sex). Frequencies and percentages were used to summarise categorical variables according to acute, serious head injury or no acute, serious head injury (i.e., sex, witnessed/unwitnessed, fall height, head strike, new symptoms 4 h post-fall, whether taking blood thinners, and blood thinner type).

### 2.4. Ethical Considerations

This project complied with the National Statement on Ethical Conduct in Human Research [13] and was in full conformance with principles of the Declaration of Helsinki (version 2013). The study was approved by the Human Research Ethics Committee as a quality assurance initiative, exempting it from full ethics committee review [13]. Digital and paper format data were stored on a secure hospital server and in a private medical office and were accessed by research personnel only.

## 3. Results

### Sample Characteristics

Eight hundred and seventy-four falls meeting inclusion criteria were identified over the 2.5-year study period. Of these, 19.1% (*n* = 167) were repeat events (i.e., patients who experienced more than one fall during the same admission), yielding 707 separate admissions with falls. This was from a total of 55,414 admissions that met the criteria from the same time frame, meaning that falls occurred in 1.3% of admissions. Most falls were unwitnessed (*n* = 676, 77.3%), and they most commonly occurred from standing height (*n* = 420, 48.1%). See Table 1.

The mean age of the people who fell was 80.4 years (SD = 7.7), and 64.9% (*n* = 567) were males. The prevalence of falls increased with age (0.68%, *n* = 74, in the 65–69 years age group; 2.22%, *n* = 21, in the 95+ age group). The females who fell were significantly older than the males who fell (81.36 years vs. 79.8 years, respectively, *p* = 0.004). Over 60% (*n* = 529) of the people who fell were delirious prior to their fall, and the vast majority had had a fall risk assessment completed before their fall (*n* = 846, 96.8%). The mean length of the stay for the people who fell was 22.77 days (IQR = 28), and 19.1% (*n* = 167) were readmitted within 30 days of discharge. Same-admission mortality was 9.3% (*n* = 84). See Table 1.

The In-Patient Post Fall Clinical Pathway was started for all falls. The vast majority of falls (*n* = 792, 90.6%) met the In-Patient Post Fall Clinical Pathway criteria for a CT head scan. Only 50.13% (*n* = 397) of patients who -met the criteria for a scan actually had a scan performed. Therefore, about half (*n* = 406, 46.5%) of all people who fell had a scan performed. See Table 1.

Acute, serious head injuries were relatively uncommon, being identified in 5.5% of scanned people who fell or 2.25% of the total people who fell (*n* = 23). The most common acute, serious head injury was subdural haematoma (*n* = 13), followed by subarachnoid haemorrhage (*n* = 5). Six people who fell had a skull, facial, or cervical spine fracture. Non-serious head injuries (e.g., scalp haematomas) were more common (*n* = 28, 3.2%), as were new CT findings not attributable to the fall (such as the expected evolution of a stroke; 4.3%, *n* = 38).

Only 0.11% (*n* = 1) of the people who fell underwent neurosurgical intervention. Another person who fell was transferred to another facility for neurosurgical monitoring but did not undergo any surgical intervention. We did not identify any cases of missed acute, serious head injury upon the review of discharge summaries and subsequent admissions within 30 days of discharge.

There were no characteristics that were significantly predictive of or associated with acute, serious head injury. Characteristics that showed a trend towards the expected association included an unwitnessed fall, a fall from bed, a fall with a head strike, new symptoms four hours post-fall, and a fall while receiving anticoagulation treatment.

## 4. Discussion

Falls are common among hospitalised older adults, with a prevalence of 1.3% in the studied health service. This is similar to the 3.6% prevalence reported in the literature based on United Kingdom data [14]. It was challenging to find comparable Australian data, as most bodies report on the prevalence of inpatient falls resulting in injury, rather than total fall numbers. The prevalence of inpatient falls resulting in injury in Australia for all age groups is 0.5% for public hospitals [2]. This rises to 0.7% for adults aged over 65 years [2].

Inpatient falls are more common with increasing age, and they showed a trend towards being more common among males, with both of these findings being consistent with previous studies identifying these as risk factors for falling [15,16]. The majority of people who fell were delirious preceding their fall, and almost 20% of falls were repeat events (i.e., patients having more than one fall in the same admission). Again, this is consistent with the previous literature identifying delirium and previous falls as risk factors for falling [17]. At the study sites, delirium is typically assessed using the 4AT [18] or the Confusion Assessment Method (CAM) [19], supported by routine nursing observations. Given its strong association with falls risk, improving delirium recognition and management is essential in fall prevention.

A minority of falls resulted in acute, serious head injury, with a prevalence of 2.3%, consistent with the literature [4]. The most common serious head injury sustained was subdural haematoma, followed by subarachnoid haemorrhage. Only one patient underwent neurosurgery for their injury, with one other being transferred to another facility for neurosurgical monitoring.

Almost half of the people who fell received a CT head scan post-event (46.5%). This is higher than the corresponding number for a recent Australian audit done in Victoria, which showed that they scanned 20% of their patients who had experienced an inpatient fall [5]. Notably, this Victorian study included adults of all ages who fell (not just older adults), and increasing age is associated with an increased risk of acute, serious head injury and a lower threshold for receiving a scan [5]. It may also reflect regional variation in practice and clinical guidelines.

With regards to the use of the In-Patient Post Fall Clinical Pathway, the vast majority of studied people who fell met the pathway criteria for a CT head scan (90.6%), largely driven by the high proportion of unwitnessed falls (77.3%). Only half of these patients actually had a scan performed (50.1%). From review of case notes, data collectors found that this was usually because the assessing clinician felt that a CT head scan was unlikely to change management, regardless of the findings, or that, though a fall was unwitnessed, the patient had denied a head strike and was felt to be a reliable historian. Our interpretation is that, although clinical tools are useful, clinical acumen was used in many cases. Notably, we did not identify any cases of missed acute, serious head injury upon a review of discharge summaries and subsequent admissions within 30 days of discharge. Of course, it is possible that some missed events went undetected completely.

The use of clinical discretion, especially when assessing reliable patients with no concerning symptoms post-unwitnessed fall, may reflect a more nuanced, context-sensitive approach to care. There is a risk of the overuse of CTs if strict pathway adherence were enforced. Our findings suggest that, perhaps, the current version of the In-Patient Post Fall Clinical Pathway has high sensitivity but low specificity, with a limited positive predictive value for serious head injury.

No analysed characteristics were significantly predictive of or associated with acute, serious head injury, again reflecting the complexity in such decision-making. The characteristics that showed a trend towards the expected association included an unwitnessed fall, a fall from bed, a fall with a head strike, new symptoms four hours post-fall, and a fall while receiving anticoagulation. All of these are already reflected in the current iteration of the Post-Fall Clinical Pathway and comparable pathways developed for the emergency and outpatient spheres [6,10].

Further investigation is required to determine whether these trends are significant; however, the identification of a fall from bed as a risk factor is particularly amenable to harm mitigation strategies. Prioritising low beds and crash mats for those identified as having a high fall risk may be of added value.

Inpatient falls are often considered preventable “never events”, [20] and fall-related injuries may impact hospital liability and funding. This may lead to increased caution in patient mobility, potentially resulting in decreased ambulation, deconditioning, and, paradoxically, increased fall risk. Future research should explore how such policies influence fall prevention and patient outcomes.

### Strengths and Limitations

The strengths of this clinician-initiated study are that it included data across two regional hospitals. It is standard practice that all fall events are reported at these sites, so, by utilising risk management software for case identification, it is hoped that all falls that met the inclusion criteria were captured. In reality, unfortunately, it is likely that a proportion of falls were not reported.

This project is limited in that it utilised a retrospective research design and constituted a review of medical records recorded by others. The risk of bias was mitigated by using trained data collectors and an adapted medical audit tool [12]. Decisions as to which risk factors for acute, serious head injury were assessed were based on current post-fall CT head scan guidelines, and additional potentially significant risk factors may have been missed. The project failed to identify significant predictors of acute, serious head injury, and though the sample was large, perhaps this potential oversight was simply due to an insufficient sample size.

Although fall risk assessments were universally completed, this audit did not explore the association between risk scores and injury outcomes. Future studies could assess whether repeated or dynamic risk assessments post-fall might better predict adverse outcomes.

## 5. Conclusions

The present project established the prevalence of inpatient falls among older adults at two regional hospitals, as well as the prevalence and nature of acute, serious head injuries sustained as a result of these events. It assessed the use of CT head scan post-inpatient fall among older adults and the adherence to Queensland’s In-Patient Post Fall Clinical Pathway. Clinical tools help guide the decision to perform a CT head scan, but they were not followed in half of cases in the studied health service. No analysed characteristics were significantly predictive of acute, serious head injury. Characteristics that showed a trend towards the expected association included an unwitnessed fall, a fall from bed, a fall with a head strike, new symptoms four hours post-fall, and a fall while receiving anticoagulation treatment. No adverse events were identified among patients who did not undergo CT head scanning.

When deciding whether a person who fell received a CT head scan, clinical acumen was used in many cases, reflecting the complexity in such decision-making, especially when assessing reliable patients with no concerning symptoms post-unwitnessed fall. The characteristics that showed a trend towards the expected association with acute, serious head injury may warrant more emphasis in the development of future decision-making tools. Further investigation is needed to inform evidence-based practice.

## Figures and Tables

**Table 1:** Sample characteristics (*n* = 874 falls).

Variable	Number (%)
**Sex**	
Male	567 (64.9)
Female	307 (35.1)
**Recurrent fall event**	
Single or first fall during admission	707 (80.9)
Recurrent fall during admission	167 (19.1)
**Fall witnessed or unwitnessed**	
Witnessed	198 (22.7)
Unwitnessed	676 (77.3)
**Fall height**	
Standing	420 (48.1)
Sitting	125 (14.3)
Out of bed	143 (16.4)
Unknown	186 (21.2)
**Head strike**	
Head strike	216 (24.7)
No head strike	413 (47.3)
Unknown	245 (28)
**In-Patient Post Fall Clinical Pathway started**	
Yes	874 (100)
No	0 (0)
**Met In-Patient Post Fall Clinical Pathway criteria for CT head scan**	
Yes	792 (90.6)
No	82 (9.4)
**CT head scan**	
CT scan done	406 (46.5)
CT scan not done	468 (53.5)
**CT head scan findings**	
Acute fracture (skull, facial, cervical spine)	6 (0.7)
Acute traumatic intracranial haemorrhage (SAH, SDH)	18 (2.1)
Acute scalp haematoma	28 (3.2)
New finding but not attributable to Fall	38 (4.3)
No new findings	310 (35.5)
**Fall risk assessment completed prior to fall**	
Yes	846 (96.8)
No	28 (3.2)
**Delirium present prior to fall**	
Yes (documented)	377 (43.1)
Probable (notes suggestive but “delirium” not mentioned explicitly)	152 (17.4)
No	345 (39.5)
**eGFR**	
≥30	788 (90.2)
15–29	70 (8)
<15	16 (1.8)
**Platelet count**	
≥100	848 (97)
50–99	15 (1.7)
<50	11 (1.3)
**Blood thinners (antiplatelet or anticoagulant)**	
Yes	477 (54.6)
Aspirin	250 (28.6)
Other antiplatelet agent (clopidogrel)	27 (3)
Anticoagulation (DOAC, warfarin, heparin infusion, therapeutic enoxaparin)	200 (23)
No	397 (45.4)
**Readmission within 30 days**	
Yes	167 (19.1)
No	707 (80.9)
**Death during admission**	
Yes	84 (9.6)
No	790 (90.4)

## Data Availability

The original contributions presented in this study are included in the article.
